# Designing nonlinearity in a current-starved ring oscillator for reservoir computing hardware

**DOI:** 10.1038/s41598-025-16209-9

**Published:** 2025-10-01

**Authors:** Nyi Nyi Tun, Shiyuan Sun, Tetsuya Iizuka, Takeaki Yajima

**Affiliations:** 1 Department of Electrical and Electronic Engineering, Graduate School and Faculty of Information Science and Electrical Engineering, Kyushu University, Fukuoka-shi, Fukuoka, 819-0395 Japan; 2 Systems Design Lab, School of Engineering, The University of Tokyo, Bunkyo-ku, Tokyo, 113-0032 Japan

**Keywords:** Nonlinear, Current-starved ring oscillator, Reservoir computing, Frequency, Learning, Energy science and technology, Engineering, Physics

## Abstract

In building spiking neural networks for edge devices, low power consumption and time scale matching with the input signal are essential characteristics for their analog implementation. In each node of the neural network, an activation function should be implemented to achieve nonlinearity between input spike frequency and output spike frequency. However, the conventional analog implementation often achieves nonlinearity in the voltage domain rather than in the spike frequency domain and consumes considerable power. In this study, a nonlinear frequency-conversion circuit based on a current-starved ring oscillator is proposed. In order to design nonlinearity in the frequency domain, the supply current for the ring oscillator is controlled as a function of input spike frequency. As a result, a hyperbolic-tangent nonlinearity is achieved in the simulation with the TSMC 180 nm process. Furthermore, the supply current is controlled in an extremely low range to achieve low power consumption of 0.2 nW and several hundred millisecond time constants, which are suitable for processing data with similar time scales such as biomedical data, environmental vibration, and so on.

**Supplementary Information:**

The online version contains supplementary material available at 10.1038/s41598-025-16209-9.

## Introduction

Reservoir computing is one of the recurrent neural network (RNN) algorithms that is expected to be used to implement learning functions in edge devices. This is because the computational cost in reservoir computing is much smaller than conventional RNN and can meet the strict power limitation in edge devices. As an RNN algorithm, reservoir computing can learn and infer time-series data. For example, it can be used to compensate for signal distortion in wireless communications^1^ adaptively or to predict blood glucose levels in real-time to prevent hyperglycemia or hypoglycemia^2^. In its algorithm, the input time-series data is processed inside the reservoir network, where each node sums up data from reservoir input and other nodes, performs a nonlinear conversion, and transfers the result to reservoir output and other nodes. Unlike general RNNs, the connection weights inside the reservoir are fixed and not the training targets, which enables training with low computational complexity.

Reservoir computing was originally proposed as the Echo State Network (ESN) and Liquid State Machine (LSM) algorithms^3,4^, which have been implemented in general-purpose computers made of digital circuits. On the other hand, reservoir computing has also been implemented in analog systems utilizing circuits, devices, and materials^5–9^. However, the conventional analog implementation often achieves a nonlinear conversion process in the voltage domain, leading to nontrivial power consumption^10^. The analog implementation by using spikes, just like the biological neural network, may provide a great opportunity to address the issue^11^.

Spiking Neural Networks (SNNs)^12–16^ have been applied to many studies in visual processing^17,18^, image processing^19,20^, speech recognition^21,22^, and medical diagnosis^23,24^ for low power consumption. In neuromorphic computing, SNN hardware implementation can be mainly classified into two categories: large-scale accelerators, such as SpiNNaker^25^, TrueNorth^26^, Neurogrid^27^, Loihi^28^, and BrainScaleS^29,30^; and small-embedded neuromorphic platforms for edge applications^14,31–33^. While the practical benefit of using spikes is still controversial, a great prospect of SNN is implied by the biological systems for lower power and higher performance than conventional digital implementation.

There are still rising challenges for the spiking reservoir computing hardware. First, the former analog circuits’ power consumption with Complementary Metal-Oxide-Semiconductor (CMOS) technology has not reached a minimum level in the order of nanowatts^34–37^, and this prevails as a research problem to be tackled for upcoming analog circuit designs^5^. Secondly, extraction of the temporal characteristics in the millisecond-to-second time scale is essential for SNNs in edge devices because this time scale is in the same order as the biological synapses and could be readily applied for biomedical data or signals from the surrounding environment. However, there were very few studies on the transient time constant of the analog circuit from the perspective of temporal data extraction. Finally, few studies have explored the design of nonlinear frequency conversion in a current-starved ring oscillator for sub-nW reservoir computing hardware. Thus, our study mainly considers the above-mentioned critical issues in designing innovative nonlinear reservoir computing hardware for solving the rising challenges.

We notably propose a current-starved ring-oscillator-based reservoir node for generating the time domain signals with 180 nm CMOS technology based on the previously published studies^38,39^. The study aims to design a circuit that performs nonlinear frequency-to-frequency conversion in sub-nW power consumption with CMOS-based analog circuits. In particular, hyperbolic tangent-type nonlinearity is designed because it is the most common form of nonlinearly in reservoir computing. In addition, transient time constant values were extracted from the designated input spike frequency range for the purposes of temporal information processing in analog SNNs. The novelty is its extremely low power consumption based on CMOS analog technology in comparison with the previous studies^40–42^. In this paper, we first explain the construction and basic operation of the current-starved ring oscillator CMOS analog circuit, which was constructed for the nonlinear frequency conversion. Then, the results of the designed nonlinear hyperbolic tangent in the frequency domain and its power consumption are explained. After that, the transient time constant values are investigated to explore the SNN’s spike-time nature and temporal signal communication. Finally, the discussion and conclusion are made at the end of the paper.

## Current-starved ring oscillator

**Fig. 1:**
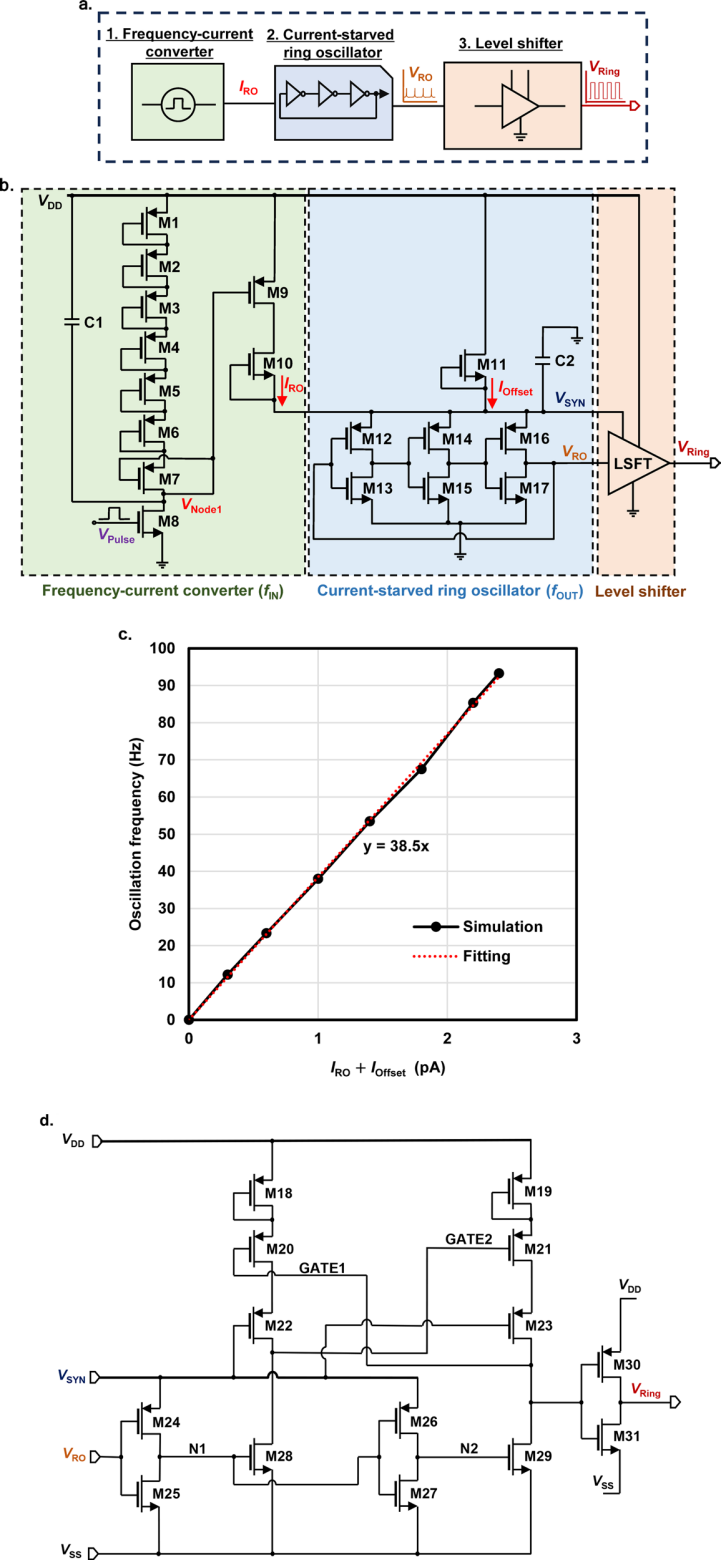
Designed a nonlinear spike-frequencyconversion circuit with a current-starved ring oscillator. (**a**) Block diagram. (**b**) Circuit diagram. (**c**) Linear characteristic of the output frequency ($\:{f}_{\text{O}\text{U}\text{T}}$) versus the supply current to the ring oscillator ($\:{I}_{\text{R}\text{O}}+\:{I}_{\text{O}\text{f}\text{f}\text{s}\text{e}\text{t}}$). (d) Level shifter circuit.

The current-starved ring oscillator (RO) is constructed to design nonlinearity in the frequency domain based on the previous study’s synapse module^38^. Figure 1a shows a block diagram of the proposed circuit. It is mainly built with three components: a frequency-current converter^38^, a current-starved RO^35,41^, and a level shifter circuit^42^. The pulse voltage ($\:{V}_{\text{P}\text{u}\text{l}\text{s}\text{e}}$) with the input spike frequency ($\:{f}_{\text{I}\text{N}}$) is given as an input, and the frequency-current conversion circuit outputs current ($\:{I}_{\text{R}\text{O}}$) that corresponds to the nonlinear conversion of $\:{f}_{\text{I}\text{N}}$. This $\:{I}_{\text{R}\text{O}}$ is fed into the current-starved RO, which generates an oscillating voltage$\:\:\left({V}_{\text{R}\text{O}}\right)$ with a frequency linear to $\:{I}_{\text{R}\text{O}}$. Finally, the level shifter circuit in Fig. 1d mainly maintains the desired output voltage range by increasing the amplitude of $\:{V}_{\text{R}\text{O}}$ to the power supply voltage $\:{V}_{\text{D}\text{D}}$.

The detailed schematic is shown in Fig. 1b. When the input $\:{V}_{\text{P}\text{u}\text{l}\text{s}\text{e}}$ with a frequency of $\:{f}_{\text{I}\text{N}}$ is fed to the M8 gate, the capacitor C1 is charged during the $\:{V}_{\text{P}\text{u}\text{l}\text{s}\text{e}}\:$width and slightly decreases $\:{V}_{\text{N}\text{o}\text{d}\text{e}1}$. Since $\:{V}_{\text{N}\text{o}\text{d}\text{e}1}$ is connected to the M9 gate, the slight decrease in $\:{V}_{\text{N}\text{o}\text{d}\text{e}1}$ increases $\:{I}_{\text{R}\text{O}}$. On the other hand, when the input $\:{V}_{\text{P}\text{u}\text{l}\text{s}\text{e}}$ is absent, the C1 is discharged gradually to increase $\:{V}_{\text{N}\text{o}\text{d}\text{e}1}$ and decrease $\:{I}_{\text{R}\text{O}}$. Therefore, $\:{I}_{\text{R}\text{O}}$ monotonically increases on average with the input frequency $\:{f}_{\text{I}\text{N}}$. In order to achieve a hyperbolic-tangent function between $\:{f}_{\text{I}\text{N}}$ and $\:{I}_{\text{R}\text{O}}$, the circuit has to implement a nonlinear onset of $\:{I}_{\text{R}\text{O}}$ for small $\:{f}_{\text{I}\text{N}}$, and saturation of $\:{I}_{\text{R}\text{O}}$ for large $\:{f}_{\text{I}\text{N}}$. The nonlinear onset is implemented by the insertion of voltage offset by M1-6 to the otherwise current-distortion circuit topology between M7 and M9. The saturation is implemented by limiting $\:{I}_{\text{R}\text{O}}$ by the zero-biased subthreshold current in M10. Since $\:{f}_{\text{O}\text{U}\text{T}}$ is linear to $\:{I}_{\text{R}\text{O}}$ as shown in Fig. 1c, a hyperbolic-tangent $\:{f}_{\text{I}\text{N}}$-$\:{f}_{\text{O}\text{U}\text{T}}$ relationship can be achieved.

The specific relationship between $\:{f}_{\text{I}\text{N}}$ and $\:{f}_{\text{O}\text{U}\text{T}}$ can be deduced in the following way. The diode-connected PMOSFET (M1) shows approximately exponential *I–V* characteristics.

1 $$\:I={I}_{1}\text{e}\text{x}\text{p}\left[\beta\:\left({V}_{\text{D}\text{D}}-V\right)\right]$$

$\:{I}_{1}$ is a constant coefficient, *V* is the drain voltage of M1, and $\:\beta\:$ is a parameter inversely proportional to the temperature. Since all the PMOSFETs M1-7 are identical, $\:{V}_{\text{D}\text{D}}-V$ = ($\:{V}_{\text{D}\text{D}}$ $\:-$ $\:{V}_{\text{N}\text{o}\text{d}\text{e}1}$) / *N*, *w*here the number of diode-connected PMOSFETs is chosen to be 7 (*N* = 7). Kirchhoff’s law for the M9 gate gives the $\:{f}_{\text{I}\text{N}}$-$\:{V}_{\text{N}\text{o}\text{d}\text{e}1}$ relationship under the steady state in the following way.

2 $$\:{I}_{\text{S}\text{A}\text{T}}d{f}_{\text{I}\text{N}}={I}_{1}\text{e}\text{x}\text{p}\left[\beta\:\left({V}_{\text{D}\text{D}}-{V}_{\text{N}\text{o}\text{d}\text{e}1}\right)/N\right]$$

$\:{I}_{\text{S}\text{A}\text{T}}d$ is the charge per input pulse that goes through M8. By multiplying $\:{f}_{\text{I}\text{N}}$, the channel current of M8 can be expressed as $\:{I}_{\text{S}\text{A}\text{T}}d{f}_{\text{I}\text{N}}$ on average. $\:{I}_{\text{S}\text{A}\text{T}}$ is the saturation current of M8 when the gate-source voltage equals $\:{V}_{\text{D}\text{D}}$, and $\:d$ is the pulse width of $\:{V}_{\text{P}\text{u}\text{l}\text{s}\text{e}}$. $\:{I}_{\text{R}\text{O}}$ can be expressed by an exponential function of $\:{V}_{\text{N}\text{o}\text{d}\text{e}1}$ as long as $\:{I}_{\text{R}\text{O}}$ is smaller than the subthreshold current under zero gate-source voltage in M10 ($\:{I}_{10}$).

3 $$\:{I}_{\text{R}\text{O}}={I}_{9}\text{e}\text{x}\text{p}\left[\beta\:\left({V}_{\text{D}\text{D}}-{V}_{\text{N}\text{o}\text{d}\text{e}1}\right)\right]\:\:\:\text{f}\text{o}\text{r}\:{I}_{\text{R}\text{O}}\ll\:{I}_{10}$$

Here, $\:{I}_{9}$ is a constant coefficient instead of under zero gate-source voltage in M9. As $\:{I}_{\text{R}\text{O}}$ increases, it saturates at $\:{I}_{\text{R}\text{O}}\cong\:{I}_{10}$. Substituting $\:{V}_{\text{D}\text{D}}-{V}_{\text{N}\text{o}\text{d}\text{e}1}$ in Eqs. (2) and (3) leads to the following equation. 

4 $$\:{I}_{\text{R}\text{O}}={I}_{9}{\left(\frac{{I}_{\text{S}\text{A}\text{T}}}{{I}_{1}}\right)}^{N}{\left(d{f}_{\text{I}\text{N}}\right)}^{N}\:\:\:\text{f}\text{o}\text{r}\:{I}_{\text{R}\text{O}}\ll\:{I}_{10}$$

$\:{I}_{\text{R}\text{O}}$ slowly increases from zero, accelerates with $\:{f}_{\text{I}\text{N}}$, and finally saturates when approaching $\:{I}_{10}$, leading to a nonlinear $\:{I}_{\text{R}\text{O}}$-$\:{f}_{\text{I}\text{N}}$ relationship that is similar to a hyperbolic tangent function. The oscillation frequency of the ring oscillator $\:{f}_{\text{O}\text{U}\text{T}}$ can be expressed by $\:{f}_{\text{O}\text{U}\text{T}}=\left({I}_{\text{R}\text{O}}+{I}_{11}\right)/{Q}_{\text{R}\text{O}}$, where $\:{Q}_{\text{R}\text{O}}$ denotes the charge consumption per oscillation in the ring oscillator and the level shifter, and $\:{I}_{11}$ is the subthreshold current under zero gate-source voltage in M11. $\:{Q}_{\text{R}\text{O}}$ is almost constant within the relevant frequency range as shown in Fig. 1c. Here, $\:{I}_{11}$ is added to $\:{I}_{\text{R}\text{O}}$ as an offset to activate stable oscillation even for a small $\:{I}_{\text{R}\text{O}}$. By substituting $\:{I}_{\text{R}\text{O}}$, the following $\:{f}_{\text{I}\text{N}}$-$\:{f}_{\text{O}\text{U}\text{T}}$ relationship can be obtained.

5 $$\:{f}_{\text{O}\text{U}\text{T}}=\frac{{I}_{9}}{{Q}_{\text{R}\text{O}}}{\left(\frac{{I}_{\text{S}\text{A}\text{T}}}{{I}_{1}}\right)}^{N}{\left(d{f}_{\text{I}\text{N}}\right)}^{N}+\frac{{I}_{11}}{{Q}_{\text{R}\text{O}}}\:\:\:for\:{f}_{\text{I}\text{N}}\ll\:{f}_{\text{M}\text{A}\text{X}}$$

6 $$\:{f}_{\text{O}\text{U}\text{T}}\cong\:\frac{{I}_{10}+{I}_{11}}{{Q}_{\text{R}\text{O}}}\:\:\:for\:{f}_{\text{I}\text{N}}>{f}_{\text{M}\text{A}\text{X}}$$

7 $$\:{f}_{\text{M}\text{A}\text{X}}=\frac{{I}_{1}}{{I}_{\text{S}\text{A}\text{T}}}{\left(\frac{{I}_{10}}{{I}_{9}}\right)}^{\frac{1}{N}}\frac{1}{d}$$

$\:{f}_{\text{M}\text{A}\text{X}}$ is the $\:{f}_{\text{I}\text{N}}$ where $\:{I}_{\text{R}\text{O}}\:$approaches the saturation value $\:{I}_{10}$, which is defined by $\:{I}_{\text{R}\text{O}}$ = $\:{I}_{10}$ in Eq. (4). The voltage that is applied to the ring oscillator ($\:{V}_{\text{S}\text{Y}\text{N}}$) is smoothed by C2. Finally, the output of the ring oscillator ($\:{V}_{\text{R}\text{O}}$) has an amplitude of $\:{V}_{\text{S}\text{Y}\text{N}}$, which is converted to $\:{V}_{\text{D}\text{D}}$ by the level shifter as shown in Fig. 1d. Here, the oscillation of $\:{V}_{\text{R}\text{O}}$ is converted to the voltages N1 and N2 in the same level, and then, their levels are upconverted to the oscillating voltages of GATE1 and GATE2. Finally, the volage GATE1 is converted to the *V*_DD_ level to output the oscillating voltage *V*_RING_. Table 1 summarizes all transistor parameters of the designed nonlinearity using a current-starved RO.

**Table 1:** Device parameters in the designed circuit.

Device	Width/length
$\:{\text{M}}_{\text{1,2},\text{3,4},\text{5,6},7}$	220 nm/180 nm
$\:{\text{M}}_{8}$	800 nm/2.4 μm
$\:{\text{M}}_{9}$	2 μm/500 nm
$\:{\text{M}}_{10}$	10 μm/180 nm
$\:{\text{M}}_{11}$	10 μm/200 nm
$\:{\text{M}}_{\text{12,14,16}}$	800 nm/8 μm
$\:{\text{M}}_{\text{13,15,17}}$	800 nm/16 μm
$\:{\text{M}}_{\text{18,19,30}}$	1 μm/1 μm
$\:{\text{M}}_{\text{20,21}}$	500 nm/10 μm
$\:{\text{M}}_{\text{22,23,24,25,26,27,31}}$	220 nm/180 nm
$\:{\text{M}}_{\text{28,29}}$	10 μm/180 nm
C1, C2	1 pF

**Fig. 2:**
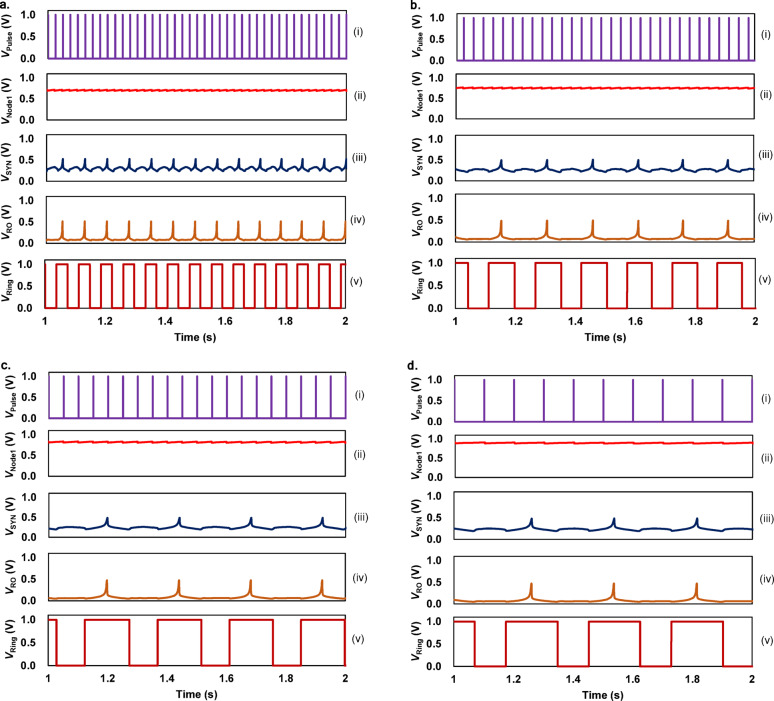
Circuit simulation for the designed circuit. (**a**–**d**) The simulated waveforms for $\:{f}_{\text{I}\text{N}}=40,\:30,\:20,\:\text{a}\text{n}\text{d}\:10\:\text{H}\text{z}$, respectively.

The simulation of $\:{V}_{\text{N}\text{o}\text{d}\text{e}1}$, $\:{V}_{\text{S}\text{Y}\text{N}}$, $\:{V}_{\text{R}\text{O}}$, and the level shifter output ($\:{V}_{\text{R}\text{i}\text{n}\text{g}}$) is shown in Fig. 2a when $\:{V}_{\text{P}\text{u}\text{l}\text{s}\text{e}}$ with $\:{f}_{\text{I}\text{N}}=40\:\text{H}\text{z}$ is fed to the M8. The simulation is based on the TSMC 180 nm Bipolar-CMOS-DMOS (BCD) process. $\:{V}_{\text{D}\text{D}}=1.0\:\text{V}$, and the width of the input spike is set to 50 ns. The simulation results for $\:{f}_{\text{I}\text{N}}$ of 30 Hz, 20 Hz, and 10 Hz are also shown in (Fig. 2b–d). As $\:{f}_{\text{I}\text{N}}$ increases, $\:{f}_{\text{O}\text{U}\text{T}}$ increases monotonically.

## Results

### Designing hyperbolic-tangent nonlinearity

**Fig. 3:**
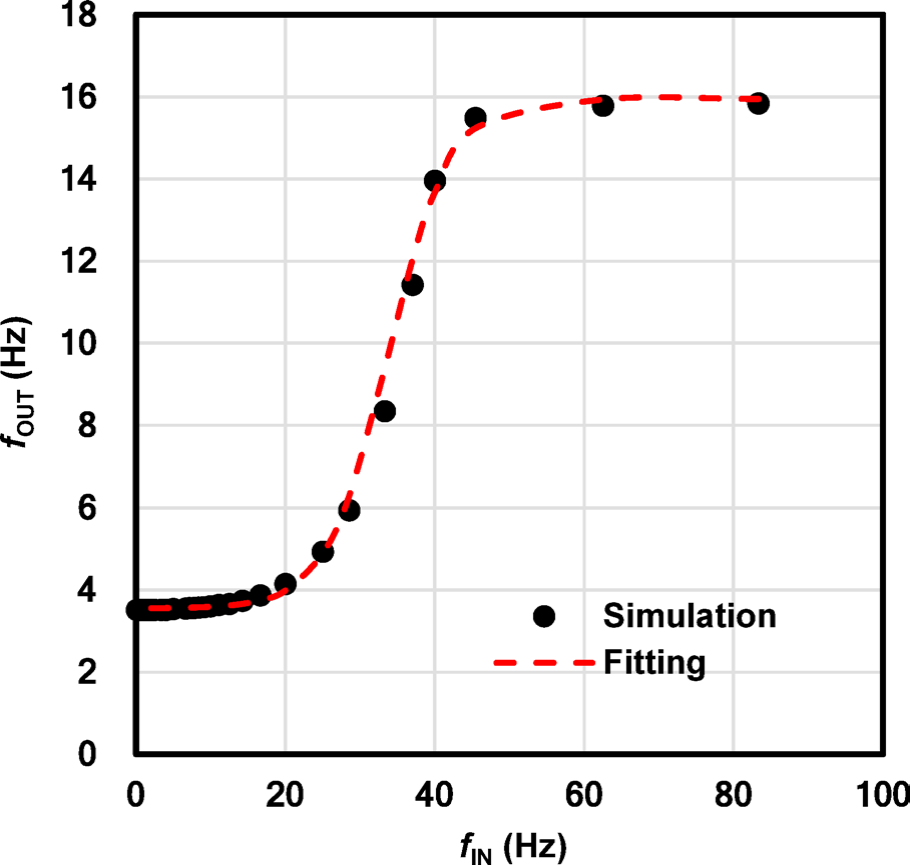
The nonlinear relationship between $\:{\varvec{f}}_{\mathbf{I}\mathbf{N}}$ and $\:{\varvec{f}}_{\mathbf{O}\mathbf{U}\mathbf{T}}$. The red dashed curve indicates the fitting curve with a hyperbolic tangent function in Eq. (8).

In analog SNNs, the nonlinear function called the activation function (AF), is critical for node design. Thus, the input-output relationship in the reservoir node is preliminarily investigated in this work. According to the simulation in Fig. 2, the relationship between $\:{f}_{\text{I}\text{N}}$ and $\:{f}_{\text{O}\text{U}\text{T}}$ in the steady state was obtained as shown in Fig. 3. Due to the nonlinear onset and the saturation in the frequency-current converter circuit, the relationship can be well fit by the hyperbolic-tangent curve, which is known to be the most standard nonlinear activation function in the reservoir node. The resulting hyperbolic-tangent curve is fitted with the equation, where K = 0.0062, a = 120, b = 0.034, and Q = 0.0098.

8 $$\:{f}_{\text{O}\text{U}\text{T}}\:=\text{t}\text{a}\text{n}\text{h}({f}_{\text{I}\text{N}}) = \text{K}\left[\frac{{(e}^{a({f}_{\text{I}\text{N}}-b)}-{e}^{-a({f}_{\text{I}\text{N}}-b)})}{{(e}^{a({f}_{\text{I}\text{N}}-b)}+{e}^{-a({f}_{\text{I}\text{N}}-b)})}\right]+\text{Q}$$

Furthermore, as transistors mainly operate in the subthreshold region, the effect of process, voltage, and temperature (PVT) variations was analyzed on the designed hyperbolic tangent relationship between the input and output frequency, as shown in Supplementary Fig. S1 and S2.

### Power consumption calculation

**Fig. 4:**
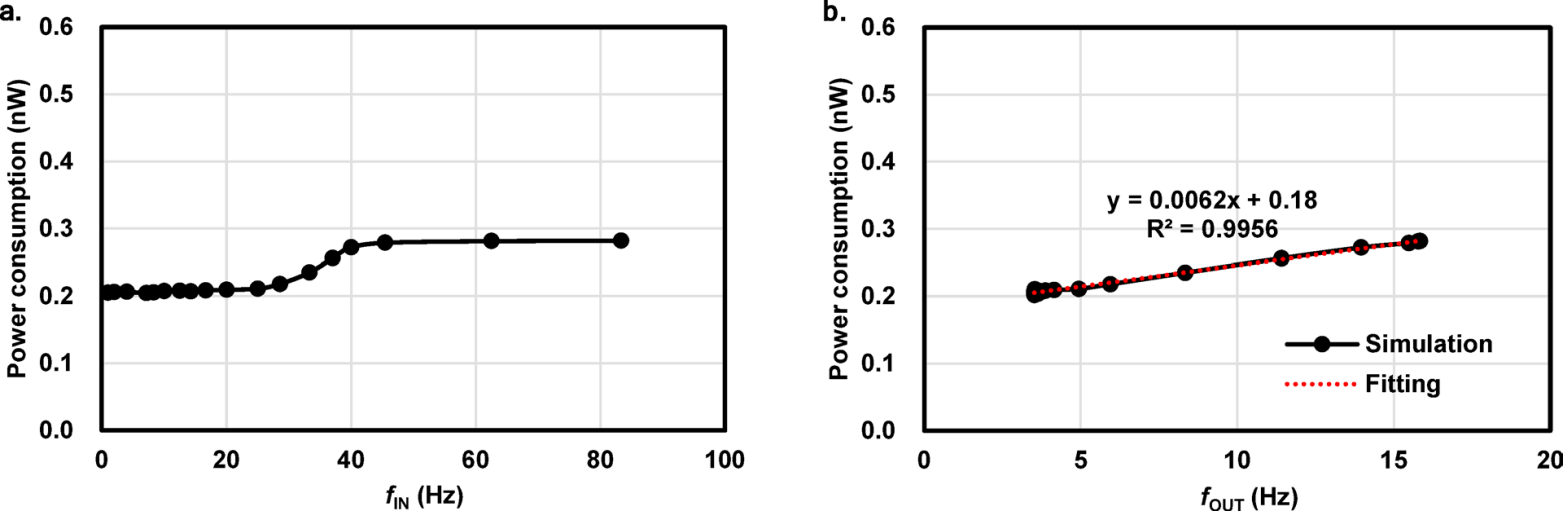
Power consumption of the designed circuit. (**a**,**b**) Power consumption as a function of $\:{f}_{\text{I}\text{N}}$ and $\:{f}_{\text{O}\text{U}\text{T}}$, respectively. The red dashed line in (**b**) is the linear fitting indicating that the power consumption is determined by the output signal generation and the constant static power.

In designing the reservoir hardware, the low power consumption must be considered. Figure 4a,b show the power consumption of the whole circuit in Fig. 1b as a function of $\:{f}_{\text{I}\text{N}}$ and $\:{f}_{\text{O}\text{U}\text{T}}$, respectively. Due to the relatively high threshold voltage (~ 0.8 V) and low leakage current for the high-voltage MOSFETs in the 180 nm process, the power consumption was found to be as small as 0.2 nW at minimum, which is the lowest ever among frequency-to-frequency conversion circuits to the best of our knowledge.

It is worth noting that the power consumption is linearly correlated to $\:{f}_{\text{O}\text{U}\text{T}}$ with the equation $\:P=0.0062{f}_{\text{O}\text{U}\text{T}}+0.18\:\left(\text{n}\text{W}\right)$, indicating the dynamic power is 6.2 pW/Hz and the static power is 0.18 nW. The power consumption amount was also checked across nine different PVT variations, as shown in Supplementary Fig. S3. The characteristics and power consumption of the current-starved RO in this work are compared with the state-of-the-art spike-conversion (neuron) circuits that were designed in simulation and experimentally fabricated in the past literature in the benchmark Table 2. The details of the PVT simulation conditions are summarized in Supplementary Table S1.

**Table 2:** Benchmark of the spike generation circuit.

Reference and publication year	Voltage (V)	Typical frequency (Hz)	Typical power (µW)	Technology (nm)	Type of input	Sim. /Meas.	$\:{{f}}_{\text{IN}}{\:-\:{f}}_{\text{OUT}}$ relationship investigation	Circuit type
^43^ 2008	3.3	MHz range	8–40	350 nm CMOS	Current	Meas.	No	Silicon neuron
^44^ 2009	3.3	**–**	0.000007 (@300 ns pulse)	350 nm AMS CMOS	Current	Sim.	No	Current-mode conductance-based SNC SFA
^45^ 2017	0.2$\:{(V}_{\text{D}\text{D}})$	1.2 ×$\:\:{10}^{3}$(@Biomimetic) 25 ×$\:\:{10}^{3}$ (@Simplified)	0.000094(@Biomimetic) 0.0001 (@Simplified)	65 nm CMOS	Current	Meas.	No	ML-based ANC
^46^ 2019	0.7	300 ×$\:\:{10}^{6}$	–	45 nm CMOS	Voltage	Sim.	No	FAS
^47 ^2021	0.5	52.6 ×$\:\:{10}^{6}$	0.58	130 nm CMOS	Voltage	Sim.	No	LIF-SFA
^39^ 2022	1	6.6	0.0000012	180 nm CMOS	Voltage	Meas.	No	IF
^38 ^2023	1	230	0.0008	65 nm CMOS	Spike	Meas.	No	LIF
^48^ 2023	0.4	30–1000	0.000249 (8.3 pJ @30 Hz) 0.000326 (326 fJ @1 kHz)	22 nm FDSOI	Current	Sim.	No	LIF
^49 ^2023	1.2	30–1000	0.01449 (483 pJ @30 Hz) 0.0184 (18.4 pJ @1 kHz)	55 nm CMOS	Current	Meas.	No	LIF-Synapse-SDSP
^50^ 2024	0.9	100 $\:\times\:\:{10}^{6}\:$– 800 ×$\:\:{10}^{6}$	0.0000001 (Low Power @0.1 V) 0.0001 (High Performance @0.1 V)	22 nm CMOS	Current	Sim.	No	LIF
^51^ 2024	0.9$\:{(V}_{\text{D}\text{D}})$	20 ×$\:\:{10}^{6}$	440	180 nm CMOS	Voltage	Meas.	No	ME-fabricated LIF
^52 ^2025	0.5	16.18 ×$\:\:{10}^{6}$	–	180 nm CMOS	Voltage	Sim.	No	CMOS-based LIF-SFA
This work	1	3.52 15.84	0.000205 (58.24 pJ per pulse) 0.000282 (17.8 pJ per pulse)	180 nm CMOS	Spike	Sim.	Yes	Current-starved RO

Simulation (Sim.), Measurement (Meas.), Input Spike Frequency ($\:{{f}}_{\text{I}\text{N}}$), Output Spike Frequency ($\:{{f}}_{\text{O}\text{U}\text{T}}$), Silicon Neuron Circuit with Spike-Frequency Adaptation (SNC SFA), Morris-Lecar-based Artificial Neuron Circuit (ML-based ANC), Frequency-Adaptive Synapse (FAS), Leaky Integrate-and-Fire Spike-Frequency Adaptation (LIF-SFA), Integrate-and-Fire (IF), Fully Depleted Silicon on Insulator (FDSOI), Leaky Integrate-and-Fire (LIF), Leaky Integrate-and-Fire-Synapse-Spike Driven Synaptic Plasticity (LIF-Synapse-SDSP), Meminductor Emulator-fabricated Leaky Integrate-and-Fire (ME-fabricated-LIF), Complementary Metal-Oxide-Semiconductor-based Leaky Integrate-and-Fire with Spike-Frequency Adaptation (CMOS-based LIF-SFA), Current-starved Ring Oscillator (Current-starved RO).

### Analysis of transient time constants

**Fig. 5:**
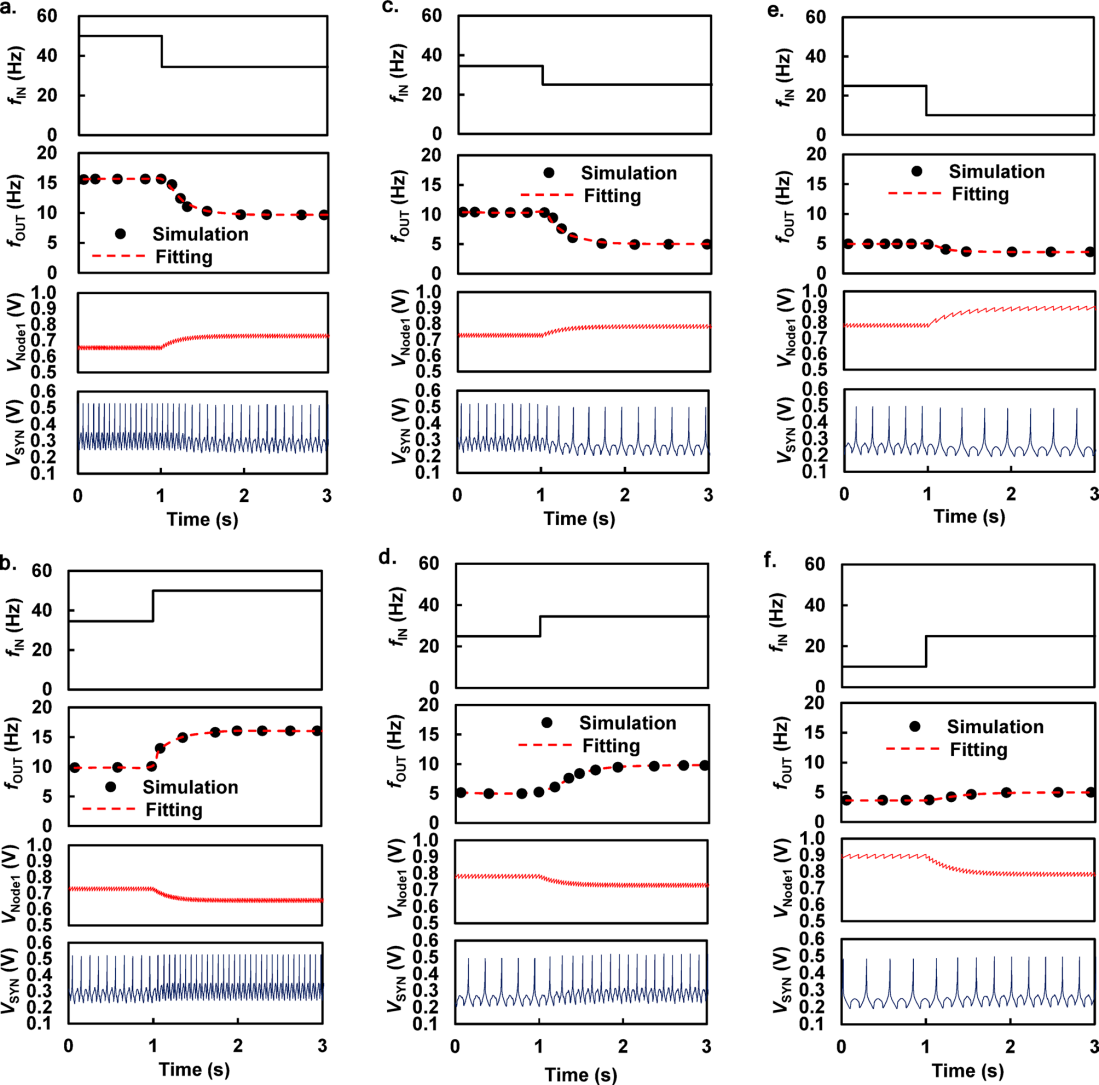
The reaction of the simulated circuit for $\:{{f}}_{\text{I}\text{N}}$ change. (**a**,**c**,**e**) $\:{f}_{\text{I}\text{N}}$ was decreased from 50 Hz to 34 Hz, from 34 Hz to 25 Hz, and from 25 Hz to 10 Hz, respectively. (**b**,**d**,**f**) $\:{f}_{\text{I}\text{N}}$ was increased from 34 Hz to 50 Hz, from 25 Hz to 34 Hz, and from 10 Hz to 25 Hz, respectively. $\:{f}_{\text{O}\text{U}\text{T}}$ was calculated from the inverse of the spike-to-spike interval. The red dashed curve indicates the exponential function, which is used to calculate the time constant in Fig. 6.

**Fig. 6:**
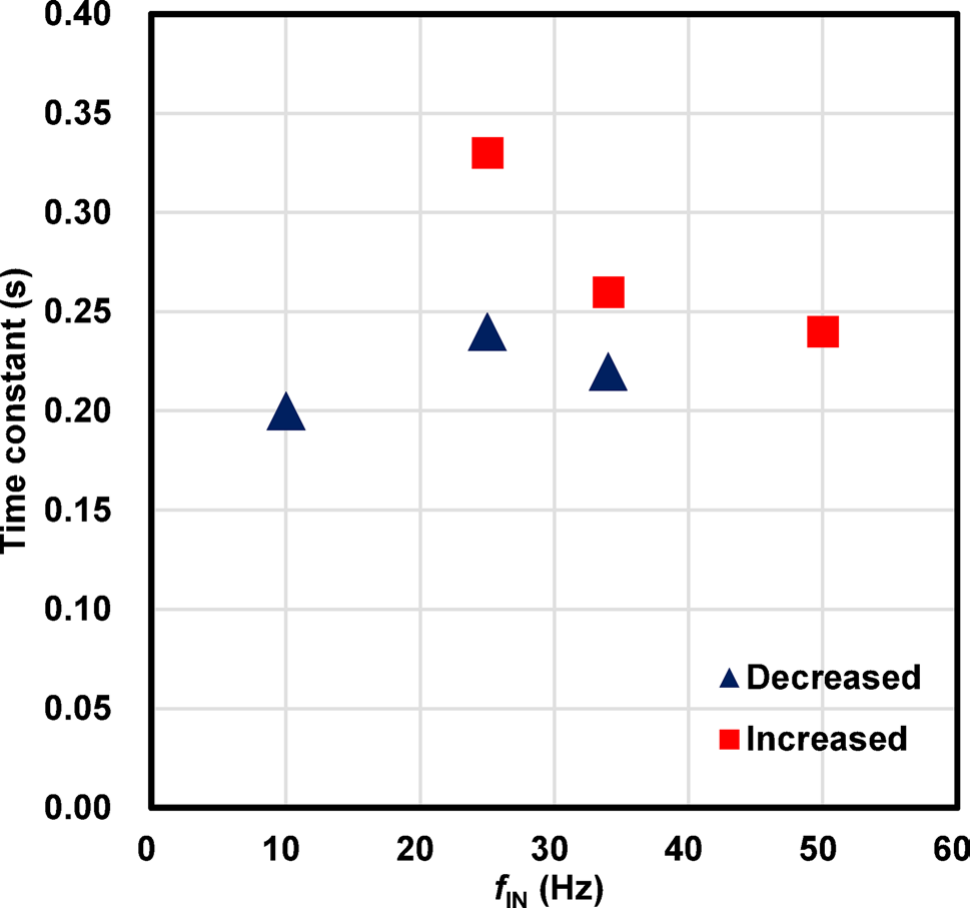
Decay time constant versus$\:\:{{f}}_{\text{I}\text{N}}$ after changing.

In the analog implementation of reservoir computing, the transient time constant in each node should be comparable to the time scale of the input time-series data for optimal performance^53^. Therefore, the transient time constant of$\:\:{f}_{\text{O}\text{U}\text{T}}$ is investigated by increasing or decreasing $\:{f}_{\text{I}\text{N}}$ discontinuously. $\:{f}_{\text{I}\text{N}}$ was changed from 50 Hz to 34 Hz (Fig. 5a), from 34 Hz to 25 Hz (Fig. 5c), from 25 Hz to 10 Hz (Fig. 5e), and vice versa (Fig. 5b,d,f), respectively. The transient curve of $\:{f}_{\text{O}\text{U}\text{T}}$ was fitted by the exponential curve to extract the transient time constant (τ). The extracted time constants are plotted as a function of $\:{f}_{\text{I}\text{N}}$ after the change in Fig. 6. The time constant is approximately 0.25 s for all the cases. This time constant in $\:{f}_{\text{O}\text{U}\text{T}}$ is mainly caused by the delay in *V*_Node1_ according to Fig. 5, which is defined by the charging or discharging time constant of C1.

## Discussion

### Nonlinear design

Here, we discuss the design principle of nonlinearity in the spike-frequency domain. To achieve hyperbolic-tangent nonlinearity, it is necessary to create a nonlinear onset on the low-frequency side and a nonlinear saturation on the high-frequency side. The nonlinear onset of $\:{f}_{\text{O}\text{U}\text{T}}$ can be achieved by simply designing a nonlinear onset in the current supply to the ring oscillator circuit. The nonlinear onset of current can be designed based on the current mirror circuit, which originally exhibits a linear relationship between the input current and the output current. When diode-connected transistors are inserted on one side of the current mirror circuit as shown by M1-6 in Fig. 1b, the input-output relationship becomes nonlinear as shown by Eq. (4). The more transistors are inserted, the more nonlinear the output current onset becomes. This current-mirror-based circuit is called the “current-distortion circuit” in this manuscript. Saturation on the high-frequency side can simply be implemented by adding a saturation function to the current-distortion circuit. By inserting a current source as shown in Fig. 1b, the current supply to the ring oscillator is made to saturate at the off current of the transistor (so-called current-starved ring oscillator). Thus, the ring oscillator and the current-distortion circuit are the two major constituents for the nonlinear spike frequency conversion. The basic design principle is as follows. The input spike frequency is converted to current by a low-pass filter (C1 in Fig. 1b), then, the current is nonlinearly processed by the current-distortion circuit, and finally, the current is converted to the output spike frequency via the ring oscillator. By modifying the design of the current-distortion circuit, other types of nonlinearity can also be achieved between $\:{f}_{\text{I}\text{N}}$ and $\:{f}_{\text{O}\text{U}\text{T}}$.

It is worth pointing out that there were a large number of previous works about the spiking neuron circuit as summarized in Table 2. However, none of these previous works have clearly shown the$\:\:{f}_{\text{I}\text{N}}$ vs.$\:\:{f}_{\text{O}\text{U}\text{T}}$ relationship even though it corresponds to a so-called “activation function” which is one of the most essential hyperparameters in the neural network algorithm. Indeed, most studies only mentioned input voltage versus output frequency such as Refs.^45–47,52^. Moreover, none of them have tried to “design” the shape of $\:\:{f}_{\text{I}\text{N}}$ vs. $\:{f}_{\text{O}\text{U}\text{T}}$ function. In this sense, this work is the first demonstration to the best of our knowledge that clearly shows the hyperbolic tangent relationship between $\:{f}_{\text{I}\text{N}}$ and $\:{f}_{\text{O}\text{U}\text{T}}$ in the spike frequency domain and its design principle based on a simple ring-oscillator scheme. Furthermore, the designed circuit consumes an extremely low-power consumption of 0.2 nW, which is a great advantage for constructing analog spiking neural network hardware.

### Application to low-power SNN

The hyperbolic-tangent nonlinearity facilitates the application of the designed circuit to SNNs and reservoir computing^10,35,46,47^. From the perspective of power consumption, the results showed a total power consumption of 0.2 nW, which is lower than most of the previous works where the power consumption lies between microwatts (µW) and milliwatts (mW)^54–57^. Only a limited work has shown a simulation result with nanowatt power consumption (5.27 nW/kHz) recently^58^. For the implementation of SNN hardware, dealing with the temporal spike signals with millisecond-to-second time scales is also critical for real-time spike signal processing and learning^11,14,16,34^. Thus, the decay time constant values were also investigated, and were shown to be around 0.25 s, which is in the same order as the biological synapses and could be readily applied for constructing SNN hardware in edge devices^15,59^.

## Conclusion

An analog implementation of a nonlinear node for SNN or reservoir computing was demonstrated. The designed circuit exhibits a hyperbolic tangent-type input-output relationship in the spike-frequency domain and achieves ultra-low power consumption of 0.2 nW and a biocompatible time constant of approximately 0.25 s. The basic design principle is based on three components: a low-pass filter for converting the input spike frequency to the current, a current-distortion circuit for specific nonlinearity, and a ring oscillator for converting the current to the output spike frequency. This ring oscillator-based design for the spike frequency conversion simplifies the system design and readily achieves ultra-low power and biocompatible time constant.

## Supplementary Information

Below is the link to the electronic supplementary material.

Supplementary Material 1

## Data Availability

The datasets used and/or analysed during the current study are available from the corresponding authors on reasonable request.
