# Supplementary material for: Designing nonlinearity in a current-starved ring oscillator for reservoir computing hardware
Source: Sci Rep. 2025 Oct 1;15:34235. doi: 10.1038/s41598-025-16209-9 (PMC12488934; doi:10.1038/s41598-025-16209-9)
Supplement: Supplementary file 1 — Supplementary Material 1 [file 41598_2025_16209_MOESM1_ESM.docx]

**Supplementary Figures and Supplementary Table**

Furthermore, as transistors mainly operate in the subthreshold region, the effect of process, voltage, and temperature (PVT) variations was analyzed on the designed hyperbolic tangent relationship between the input and output frequency, as shown in Supplementary Fig. S1 and S2.

**Supplementary Figure S1. PVT variation analysis.** Normalized $f_{\mathrm{OUT}}$ vs normalized $f_{\mathrm{IN}}$ for TT (typical), SS (Slow NMOS, Slow PMOS)**,** FF (Fast NMOS, Fast PMOS), FS (Fast NMOS, Slow PMOS), SF (Slow NMOS, Fast PMOS), T + 25 (Temperature 27 ℃ + 25℃), T – 25 (Temperature 27 ℃ – 25℃), V + 0.1 (Supply Voltage 1.0 V + 0.1 V), V – 0.1 (Supply Voltage 1.0 V – 0.1 V). The normalization was performed by the critical $f_{\mathrm{IN}}$ and the saturated $f_{\mathrm{OUT}}$ as listed in Supplementary Table S1.

**The details of the PVT simulation conditions are summarized in Supplementary Table S1.**

| **PVT conditions** | **Supply voltage (V)** | **TUNE (V)** | **TUNE LSFT (V)** | **Critical** $\boldsymbol{f}_{\mathbf{IN}}$**(Hz)** | **Saturated**$\boldsymbol{f}_{\mathbf{OUT}}$**(Hz)** |
| --- | --- | --- | --- | --- | --- |
| TT | 1.0 | 0.30 | 0 | 83.33 | 15.84 |
| SS | 1.1 | 0.30 | 0 | 7.692 | 91.58 |
| FF | 1.0 | 0.50 | 0 | 333.3 | 165.8 |
| FS | 1.0 | 0.50 | − 0.5 | 10.00 | 782.4 |
| SF | 1.0 | 0.60 | 0 | 1250 | 23.38 |
| T + 25℃ | 1.0 | 0.55 | 0 | 333.3 | 212.2 |
| T – 25℃ | 1.0 | 0.60 | 0 | 25.00 | 1.022 |
| V + 0.1 V | 1.1 | 0.55 | 0 | 100.0 | 18.16 |
| V – 0.1 V | 0.9 | 0.55 | 0 | 100.0 | 14.11 |

**Supplementary Table S1. Summary of PVT simulation conditions.** For each condition, the parameters were adjusted such as the supply voltage (*V*_DD_), the tuning voltage for the bodies of M10 and M11 (TUNE), and the tuning voltage for the bodies of M28 and M29 in the level shifter circuit (TUNE LSFT). The critical $f_{\mathrm{IN}}$ is defined at the maximum slope of $f_{\mathrm{OUT}}$ vs $f_{\mathrm{IN}}$, and the saturated $f_{\mathrm{OUT}}$ is defined at a sufficiently large $f_{\mathrm{IN}}$.

**Supplementary Figure S2. Designed a nonlinear spike-frequency conversion circuit with a current-starved ring oscillator circuit diagram with the added TUNE and TUNE LSFT for the PVT simulation analysis.**

**The power consumption amount was also checked across nine different PVT variations, as shown in Supplementary Fig. S3.**

**Supplementary Figure S3. Power consumption for PVT variations.**
